# Impacts of Host, Cell Density, and Timing of Injection on Efficiency for Xenogen Production in Ictalurid Catfish

**DOI:** 10.1007/s10126-025-10466-5

**Published:** 2025-05-27

**Authors:** Kate B. Pottle, Darshika U. Hettiarachchi, Mei Shang, Baofeng Su, Jacob Al-Armanazi, Jinhai Wang, Misha Soman, Hamza Dilawar, Ian A. E. Butts, Rex A. Dunham

**Affiliations:** School of Fisheries, Aquaculture and Aquatic Sciences, Auburn University, Auburn, Alabama 36849 USA

**Keywords:** Reproductive technology, Xenogenesis, Blue catfish, White catfish, Channel catfish, Surrogacy

## Abstract

Optimization of xenogenesis for hybrid catfish (♀ channel catfish, *Ictalurus punctatus* × ♂ blue catfish, *I. furcatus*) embryo production was the goal. The effect of density of unsorted gonadal cells (80,000, 100,000, or 120,000 cells/fry) from blue catfish (BGCs) injected into triploid channel catfish surrogates, and BGCs or channel catfish (CGCs) into triploid white catfish (*Ameiurus catus*) surrogates on proliferation and colonization rates in surrogates injected at 4-, 5-, or 6-days post-hatch (DPH) was evaluated. At 45 and 90 DPH, survival and size of surrogates, and colonization/proliferation of donor cells (cell area < 150 μm^2^ and cluster area > 150 μm^2^) were evaluated. Survival and size of all surrogate species were not impacted by cell density or donor. All surrogate species injected with 100,000 cells/fry had larger cluster cell areas than those injected with 80,000 cells/fry. White catfish surrogates with BGCs and CGCs had larger cell areas when injected with 100,000 cells/fry than those injected with 80,000 cells/fry. Both cell and cluster area increased by 90 DPH for all surrogates. PCR and PKH26 red fluorescence analysis confirmed that > 89% and > 86% of surrogates were positive xenogens at 45 and 90 DPH, respectively. No surrogate type or donor was superior to the others regarding colonization and proliferation, survival or growth, thus, channel catfish or white catfish were equally effective surrogates. Potential advantages of white catfish are small size, early sexual maturity, and spawning early in the season. These findings enhance the efficiency of germ cell transplantation for commercial hybrid catfish production.

**Supplementary Information:**

The online version contains supplementary material available at 10.1007/s10126-025-10466-5.

## Introduction

The catfish industry continues to be the market leader in the U.S. aquaculture sector, with Mississippi, Alabama, Arkansas, and Texas producing 96% of total domestic sales. Overall, catfish generated $437 million in pond bank sales in 2023 (NASS 2024). Today, hybrid catfish (♀ channel catfish, *Ictalurus punctatus* × ♂ blue catfish, *I. furcatus*) is the most commonly cultured genotype in the catfish industry (NASS 2024) due to its high fillet yield (Dunham et al. 1987), seinability (Dunham and Argue 1998), high dress out percentage (Bosworth et al. 2004), tolerance to low dissolved oxygen (Dunham et al. 1983), increased disease resistance (Arias et al. 2012; Dunham et al. 2014), efficient feed conversion ratio, and rapid, yet uniform, growth rate (Yant et al. 1976). An impediment to the industry is the labor-intensive in vitro fertilization (IVF) process that is used to create hybrid catfish. For instance, IVF entails hormonal stimulation, bag spawning, manual egg stripping of females, and sacrifice of males for sperm removal (Dunham and Masser 2012), which can take 4 to 6 years of resources to raise the males to maturity (Graham 1999). To aid in the efficiency and sustainability of creating hybrid catfish, an innovative technology known as xenogenesis is being developed (Perera et al. 2017).

Xenogenesis is a method of reproduction in which successive generations differ from each other phenotypically and genetically, and no genetic material is transmitted from the parent to the offspring (Dunham 2023). Primordial germ cells (PGCs), spermatogonial stem cells (SSCs), or oogonial stem cells (OSCs) are derived from gonads of immature donor diploid fish and then transplanted into sterile triploid recipients (Perera et al. 2017; Shang et al. 2018). This transfer leads to the development of donor-derived gametes in the surrogate (Amer et al. 2001; Yoshizaki et al. 2010; Wong et al. 2011). Since PGCs, SSCs, and OSCs, can migrate and colonize the genital ridge after transplantation, they are able to produce either ova or sperm in the surrogate depending on the surrogate’s genetic sex (Yoshizaki and Lee 2018).

Our previous studies have refined the xenogenesis technology process for hybrid catfish (Shang et al. 2015; Perera et al. 2017; Hettiarachchi et al. 2020, 2022, 2023a, b; Abualreesh et al. 2020, 2021a, b) and this technology continues to evolve to support the aquaculture industry. Past studies have improved stem cell culturing techniques and enabled SSC specific marker identification (Shang et al. 2015); used xenogenesis to successfully produce channel × blue hybrid catfish (Perera et al. 2017); found the optimal donor size for stem cell extraction (Hettiarachchi et al. 2020); created cryopreservation techniques for oogonia (Abualreesh et al. 2021a) and spermatogonia (Abualreesh et al. 2020; 2021b); produced xenogenic catfish with cryopreserved testes and ovarian tissues (Hettiarachchi et al. 2022); assessed the ideal age to inject triploid fry (Hettiarachchi et al. 2023a); and assessed the effects of seasonality on germ cell extraction (Hettiarachchi et al. 2023b).

The most recent study by Hettiarachchi et al. (2024) assessed the feasibility of using white catfish (*Ameiurus catus*) as a surrogate species to produce xenogenic catfish. Blue catfish (BGCs) and channel catfish (CGSs) donor gonadal cells were transplanted into surrogate triploid white catfish fry from 0 to 12 days post-hatch (DPH). A survival rate of > 81.2% was reported after transplantation for fry injected between 4 to 5.5 DPH, demonstrating acceptance of the foreign cells (Hettiarachchi et al. 2024). Once these white catfish surrogates mature, assessing whether they can successfully produce channel × blue hybrid catfish when pair mated will be the next landmark.

Xenogenic technology using white catfish surrogacy has several advantages. The white catfish reaches sexual maturity in only 1 to 2 years, compared to the 2 to 4 years for channel catfish, or 4 to 6 years for blue catfish (Graham 1999). Due to the shortened time to reach sexual maturity, using xenogenic white catfish to produce hybrid catfish could result in reduced feed costs, decreased holding space, and reduced labor costs (Hettiarachchi et al. 2024). Luteinizing hormone releasing hormone analogue (LHRHa) hormone implants at 90 µg/kg can also be used to induce spawning in white catfish (Fobes 2013), making this species a prime candidate as a surrogate for producing hybrid embryos in a synchronized manner (Hettiarachchi et al. 2024).

Most hybrid catfish xenogenesis transplantation studies have been conducted with channel catfish surrogates and with limited knowledge on how the density of unsorted donor gonadal cells impacts colonization and proliferation when injected into surrogates. Up to 80,000 cells/fry have been injected in both channel catfish (Hettiarachchi et al. 2020, 2022, 2023a) and white catfish surrogates (Hettiarachchi et al. 2024).

**Fig. 1:**
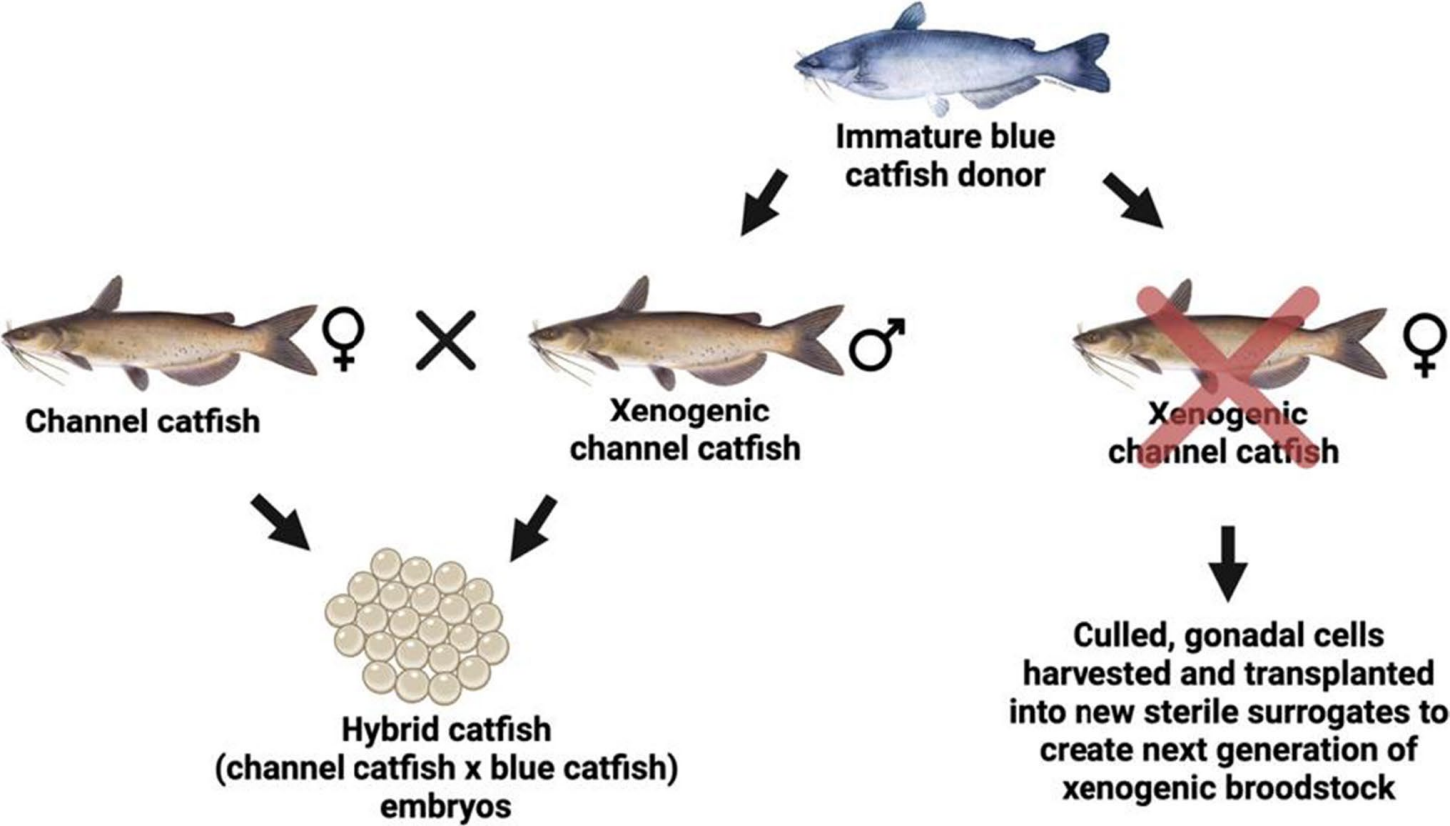
Unsorted gonadal cells extracted from immature blue catfish (*Ictalurus furcatus*) gonads and injected into triploid channel catfish (*I. punctatus*) surrogates. Channel catfish surrogates become xenogenic, and when mated with a normal channel catfish female, they can produce hybrid (♀ channel catfish × ♂ blue catfish) catfish embryos. Unused xenogenic channel catfish can be culled, and their gonads can be harvested and transplanted into new sterile surrogates to create the next generation of broodstock (Pottle 2025a, b)

Two alternative strategies are possible to make channel catfish female × blue catfish male hybrid catfish embryos. The first, a channel catfish system, is more straightforward (Fig. 1). Xenogenic channel catfish males producing blue catfish sperm are generated and mated with normal channel catfish females to produce hybrid embryos. One-half of the xenogenic individuals produced are female and can be utilized to harvest stem cells to make the next generation of xenogenic males. Thus, if desired, blue catfish are no longer needed on the farm after the first generation.

**Fig. 2:**
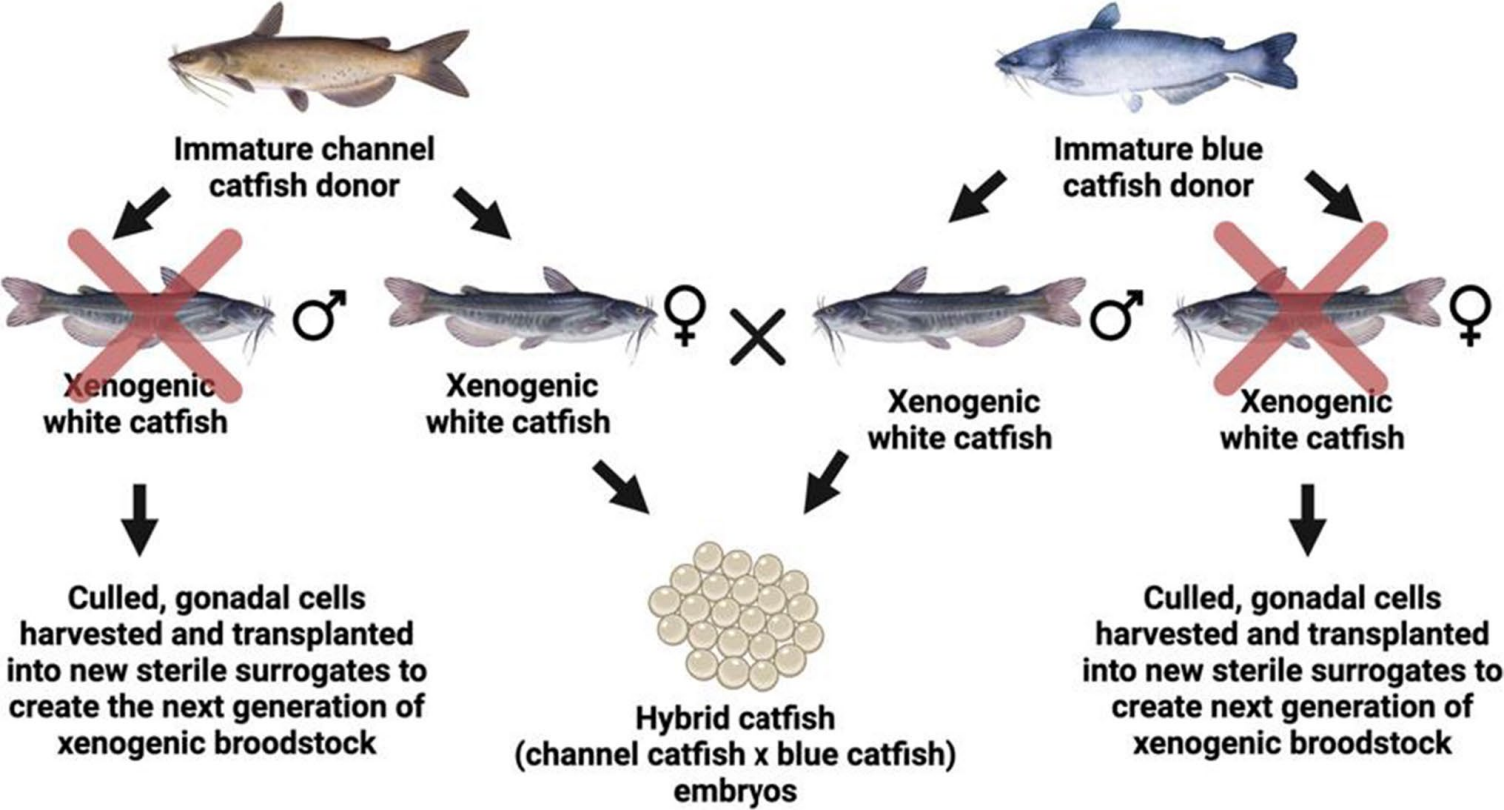
Unsorted gonadal cells extracted from immature blue catfish (*Ictalurus furcatus*) or channel catfish (*I. punctatus*) gonads and injected into triploid white catfish (*Ameiurus catus*) surrogates. White catfish surrogates become xenogenic, and when mated correctly, they can produce hybrid (♀ channel catfish × ♂ blue catfish) catfish embryos. Unused xenogenic white catfish can be culled, and their gonads can be harvested and transplanted into new sterile surrogates to create the next generation of broodstock (Pottle 2025b)

The second strategy is more complicated and utilizes white catfish as hosts (Fig. 2) with the potential advantages being the early sexual maturity of this species. Blue catfish stem cells are transferred to triploid white catfish to produce xenogenic males producing blue catfish sperm and channel catfish stem cells to produce xenogenic white catfish females producing channel catfish eggs. When these two types are mated, hybrid embryos are produced. Again, in each case, the unused sex can be utilized to generate stem cells for the next generation. Thus, after one generation, neither channel catfish or blue catfish broodstock are required for channel-blue hybrid catfish farms.

Thus, the objective of this study was to determine how the density of unsorted gonadal cells (80,000, 100,000, or 120,000 cells/fry) from BGCs into channel catfish surrogates, or BGCs and CGCs into white catfish surrogates impacts rates of proliferation and colonization when surrogates are injected at 4-, 5-, or 6 DPH. Additionally, we examined whether BGCs or CGCs are more suitable for cell transplantation into white catfish surrogates, and if there are differences between the efficiency of generating channel catfish or white catfish xenogenic hosts.

## Materials and Methods

All investigations and experimental studies on animals were conducted according to the Institutional Animal Care and Use Committee (IACUC) and the Association for Assessment and Accreditation of Laboratory Animal Care (AAALAC) protocols and guidelines for Auburn University Institutional Animal Care and Use Committee (AU-IACUC # 2021:3893).

### Broodstock Management

Broodstock were housed at the Auburn University E.W. Shell Fisheries Center in Auburn, Alabama in 2022 and 2023. Channel catfish (3- to 4-year-old) and white catfish (1.5 to 3-year-old) were cultured in 0.04-ha earthen ponds (~ 1 m in depth) and fed five days a week with 32% protein pellet feed during the summer and three days per week during the winter. Leading up to the spawning season, feed was shifted to a 36% protein broodstock feed provided five days per week. In May, sexually mature male (N = 4, mean body weight ± SEM = 0.75 ± 0.10 kg) and female white catfish (N = 4, mean body weight ± SEM = 0.70 ± 0.15 kg) were harvested from earthen ponds by seining, using a 3.8 cm mesh net. Similarly in June, mature channel catfish females (N = 4, mean body weight ± SEM = 0.80 ± 0.20 kg) and males (N = 4, mean body weight ± SEM = 0.70 ± 0.30 kg) were harvested from the earthen ponds by seining. All broodstock were stocked into 670 to 750 L flow through holding tanks using pond water at a rate of 30 L/min.

### White Catfish Spawning

Upon harvest, gravid white catfish females and mature white catfish males were administered an intraperitoneal implant of LHRHa at 90 µg/kg body weight and pairs were placed in 60 L clear glass aquaria. The pairs were left undisturbed for 36 h before each tank was observed every 4 h for signs of ovulation as indicated by a few eggs observed on the bottom of the tanks.

### Channel Catfish Spawning

Upon harvest, gravid channel catfish females were administered luteinizing hormone releasing hormone analogue (LHRHa) at 90 µg/kg body weight via intraperitoneal implantation. Following implantation, the females were placed into individual mesh bags (38 cm × 56 cm) and held ~ 30 cm apart in 670 to 750 L flow through (30 L/min) pond water holding tanks. Temperature in the tanks ranged from 26 to 28 °C. At 36 h post-implantation, bags were checked every 4 h for eggs attached to the spawning bag, which indicates that the female is ovulating.

### In Vitro Fertilization Procedures

Upon signs of ovulation, both white catfish and channel catfish females were removed from tanks and anesthetized in an 18 L bucket with 100 ppm tricaine methanesulfonate (MS-222, Syndel USA, Ferndale, WA) buffered with NaHCO_3_ to a pH of 7.0. Once anesthetized, the female fish were rinsed with pond water and dried thoroughly. Crisco® vegetable shortening was carefully rubbed on the underside of the females and eggs were hand-stripped into Crisco® coated metal pans (~ 25 g of eggs/pan).

Males (both white catfish and channel catfish, respectively) were euthanized and their sperm were collected for IVF following protocols by Dunham and Masser (2012) and Hettiarachchi et al. (2022). In brief, after euthanasia, testes were removed from the body cavity with a sterile scalpel and forceps. Testes were rinsed with 0.9% saline solution to remove any blood. After rinsing, the testes were gently dried and then minced with a scalpel blade. Following mincing, the testes were filtered with a 100 μm mesh. Thereafter, 10 mL of 0.9% saline was added for each 1 g of testes (w/v). Following filtration, the sperm solution was ready for fertilization.

### Triploid Induction

All IVF was conducted at 20 to 22 °C for white catfish and 27 to 28 °C for channel catfish. To accomplish triploid induction for embryos from both catfish species, the hand-stripped eggs were first mixed in a metal pan with freshly collected sperm (from either channel catfish or white catfish males, respectively) at a rate of 2 mL of sperm solution (10 mL of 0.9% saline per 1 g of testes) per 25 g of eggs for 2 min (Hettiarachchi et al. 2023a). Next, a fertilizing solution was prepared by adding 6 g of powdered Fullers’ Earth (MP Biomedicals, Santa Ana, CA) to 1 L of water. The fertilizing solution was mixed with the sperm and egg solution to prevent egg adhesion. After 3 min of gentle mixing with the fertilizing solution, eggs were transferred into a hydrostatic press, and at 5 min post-fertilization 7,500 psi of hydrostatic pressure was applied for 5 min (Perera et al. 2017; Hettiarachchi et al. 2022, 2023a). Following pressure shock, eggs were moved into a flow-through pond water hatching trough (supplemented with CaCl_2_ at 50 ppm) and left undisturbed for 1 h for hardening. Temperature in the hatching trough ranged from 20 to 22 °C for white catfish eggs and 26 to 28 °C for channel catfish eggs, and the flow rate was held at 3.79 L/min. After 1 h, eggs were moved into hanging mesh baskets (7.0 m × 0.4 m × 0.2 m), which were suspended in a flow-through pond water hatching trough with paddlewheel agitation and compressed aeration with a flow rate of 15 L/min.

### Isolation of Donor Stem Cells

**Fig. 3:**
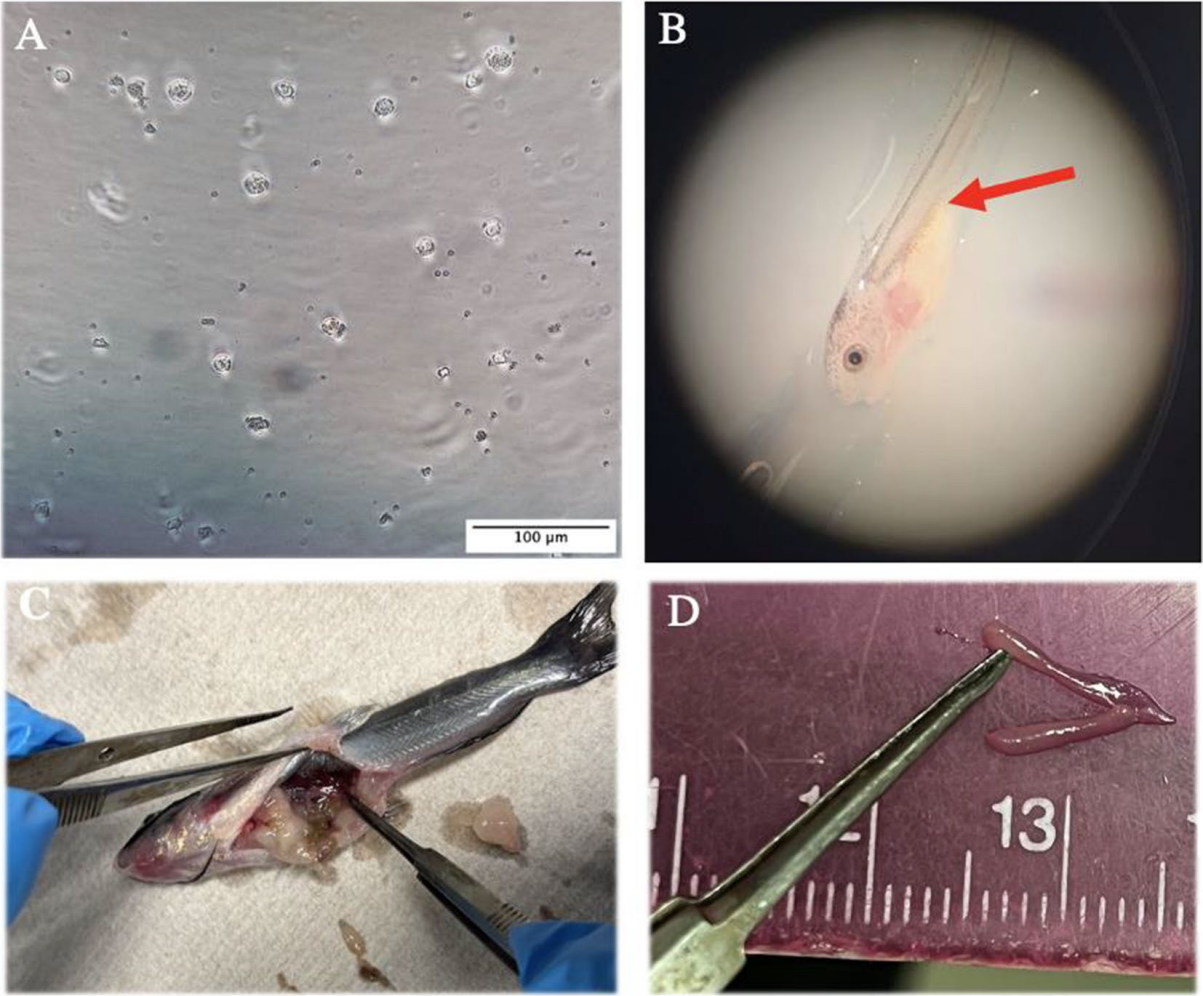
**A** Unsorted gonadal cells extracted from immature blue catfish (*Ictalurus furcatus*) gonads. **B** Injection site for transplantation (intraperitoneally) of donor derived gonadal cells into triploid white catfish (*Ameiurus catus*) or channel catfish (*I. punctatus*). Cells are then expected to migrate to the genital ridge of the recipient and initiate oogenesis or spermatogenesis. **C** Gonad removal from surrogate channel catfish fingerling. **D** Surgically removed gonad from female triploid channel catfish surrogate fry at 90 days post-hatch

Prior to hatching of triploid embryos, sexually immature (1- to 2-year-old) channel catfish (N = 6 [3 male and 3 female], mean total body length ± SEM = 30.9 ± 2.2 cm, mean total body weight ± SEM = 299.6 ± 69.9 g) and blue catfish (N = 12 [6 male and 6 female], mean total body length ± SEM = 33.6 ± 2.0 cm, mean total body weight ± SEM = 357.4 ± 79.9 g) were harvested from earthen ponds with a 3.8 cm seine net and kept in a 670 to 750 L flow-through pond water holding tank. Once white catfish eggs had hatched and fry reached 4-, 5-, and 6-DPH, channel catfish and blue catfish donors were selected and euthanized. Similarly, once channel catfish eggs had hatched and fry reached 4-, 5-, and 6-DPH, blue catfish donors were selected and euthanized For each host species, one male and one female of the corresponding donor species were sacrificed for each injection day and fresh ovarian and testicular cells mixed for injection. Following euthanasia, gonad removal and gonadal cell extraction was performed for both BGCs and CGCs following the methods of Shang et al. (2015), Abualreesh et al. (2020, 2021a, b), and Hettiarachchi et al. (2020, 2022, 2023a, b, 2024). The protocol resulted in a cell suspension comprised of unsorted gonadal cells including SSCs, OSCs, and PGCs (Fig. 3A).

### Labeling Unsorted Gonadal Cells and Injection

Following gonadal cell extraction and isolation, BGCs and CGCs were labeled with PKH26 red fluorescence cell linker (CGLDIL, Sigma-Aldrich, St. Louis, MO) according to manufacturer’s instructions. Unsorted cells for both BGCs and CGCs were counted using a hemocytometer and divided into nine separate tubes: 80,000, 100,000, and 120,000 stem cells/μL for BGCs (× 2, one set for white catfish surrogates, one set for channel catfish surrogates) and 80,000, 100,000, and 120,000 stem cells/μL for CGCs (× 1, all for white catfish surrogates). Attempts were made to transplant each stem cell quantity into triploid white catfish and channel catfish fry at 4-, 5-, and 6-DPH following the standard protocols (Hettiarachchi et al. 2023a, b, 2024). Hettiarachchi et al (2023a, 2024) examined 0–12 DPH for injection of stem cells in two separate studies, and found that 4, 5, and 6 DPH gave peak survival and percent xenogens for both channel catfish and white catfish surrogates. The 120,000 cells/fry treatment group ultimately failed due to viscosity and was removed from further analyses.

Cell transplantation began by anesthetizing white catfish surrogates (2 donor species × 2 cell density concentrations × 3 injection days × 3 replicates × 20 fry = 720) and channel catfish surrogates (1 donor species × 2 cell density concentrations × 3 injection days × 3 replicates × 20 fry = 360) with 10 mg/L tricaine methanesulfonate (MS-222) buffered with 10 mg/L sodium bicarbonate solution. Fry were placed in a petri dish (100 mm × 15 mm), observed under a microscope at 1.5 × (Amscope, Irvine, CA), and manually microinjected (Hamilton, Reno, NV). For injections, 1 μL of unsorted cell suspension (BGCs or CGCs) containing either 80,000 or 100,000 cells were injected with a 33-gauge needle (outer diameter: 0.209 mm; inner diameter: 0.108 mm; Hamilton, Reno, NV). The needle was inserted in the region where the genital ridge was expected to form; the cavity between the anal fin and yolk sac (Fig. 3.B) (Hettiarachchi et al. 2023a, 2024). An additional replicate of 20 non-injected fry for each injection day was used as the control. Following injection, treated and control fry were moved to mesh cages (0.2 m × 0.2 m × 0.25 m) in aerated pond water flow-through hatching troughs (flow rate: 15 L/1 min) at a density of 20 fry/cage.

The transplantation resulted in three surrogate types: channel catfish surrogates with BGCs (Fig. 1), white catfish surrogates with BGCs (Fig. 2), and white catfish surrogates with CGCs (Fig. 2).

### Survival and Growth

Following a 12-h recovery period, all fry were fed a standard commercial catfish fry feed (crude protein: 50.00%, crude fat: ≥ 4.00%, crude fiber: 7.00%, and phosphorus: 0.80%) 4 to 6 times per day to satiation. Feed pellet size was gradually increased to accommodate fry growth (Hettiarachchi et al. 2020, 2023a, 2024). Two sampling intervals, 45 and 90 DPH, were used to record fry survival, growth, PKH26 fluorescence, and genomic DNA/PCR data. At both sampling intervals fry survival data were collected by counting remaining fry, and 9 fingerlings were randomly sampled per replicate from each treatment (27 total per treatment) to determine total length (TL) and body weight (BW) at each time interval.

### PKH26 Observations

**Fig. 4:**
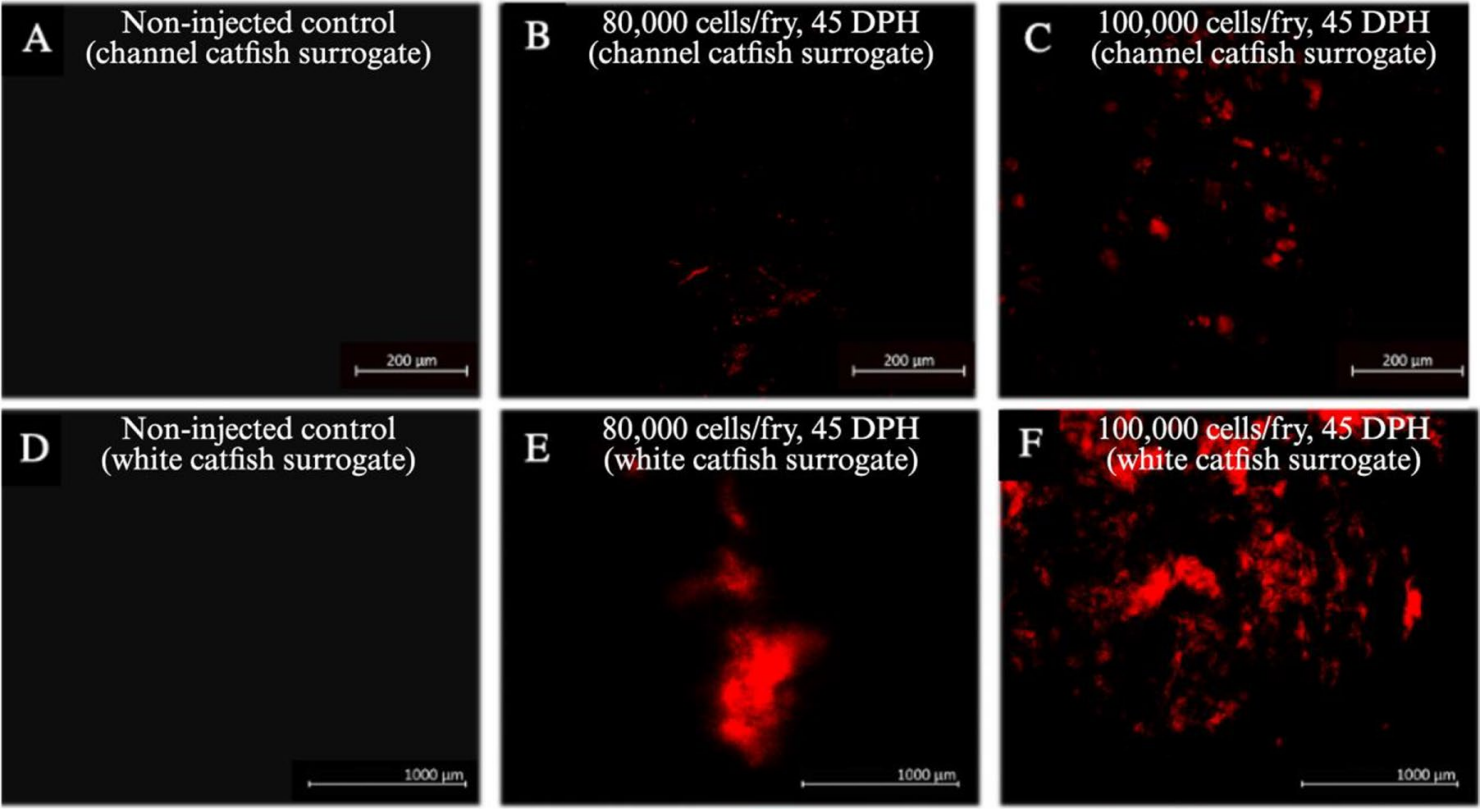
Gonadal tissues of a triploid channel catfish (*Ictalurus punctatus*, surrogate; A-C) and white catfish (*Ameiurus catus*, surrogate; D-F) expressing fluorescence from PKH26 dyed donor-derived unsorted gonadal cells. (**A**&**D**) A non-injected control treatment sampled at 45 days post-hatch (DPH), showing no fluorescence. (**B**&**E**) Cell/cluster areas in a triploid channel catfish or white catfish (surrogates), respectively, sampled at 45 DPH injected with 80,000 blue catfish (*I. furcatus*) unsorted gonadal cells/fry at 5 DPH. (**C**&**F**) Cell/cluster areas in a triploid channel catfish or white catfish (surrogates), respectively, sampled at 45 DPH injected with 100,000 blue catfish unsorted gonadal cells/fry at 5 DPH

To evaluate colonization and proliferation of BGCs and CGCs in triploid white catfish (mean total BW range = 4.53—5.53 g at 45 DPH and 7.28—8.22 g at 90 DPH) and BGCs in channel catfish surrogates (mean total BW range = 3.81—4.81 g at 45 DPH and 6.83—8.03 g at 90 DPH), 3 fingerlings were randomly selected from each treatment (4, 5, and 6 DPH, 80,000 vs. 100,000 cells), sacrificed, and their gonads were surgically removed (Fig. 3C). Each gonad (Fig. 3D) was placed on a sterile microscope slide (Life Technologies, Carlsbad, CA) and observed microscopically. Digital images were taken using a Zeiss Imager A2 microscope equipped with a digital camera (Axio-cam 202) and Zen Pro v.6.1 software (Zeiss, Oberkochen, Germany). If a sample had a fluorescing region, it was deemed positive with transferred cells, while if no fluorescing region was detected, it was deemed negative. Three fluorescent images were taken of each positive gonad sample and further analyzed using Image J software. In Image J, fluorescing regions were measured, where cell areas had a fluorescence area < 150 μm^2^ and cell cluster areas had a fluorescence area > 150 μm^2^ (Fig. 4). These area designations were chosen as stem cells can migrate individually or as clusters (Wu and Lin 2011) and collective cell migration of these clusters is the most common type of migration during development (Trepat et al. 2012). Cell–cell adhesion and communication is important for proper migration and polarity (Barriga and Mayor 2015). Singularly and collectively, the fluorescing areas should represent colonization and proliferation of the microinjected donor cells.

### Genomic DNA/PCR Analysis

Following PKH26 analysis, diagnostic assays were run to determine if sampled gonads had blue catfish or channel catfish DNA, respectively. Sampled gonads were placed into 1.5 mL Eppendorf tubes and held on ice and at −80 °C until DNA extraction. Here, samples were digested using proteinase K, followed by protein and ethanol precipitation for DNA extraction. PCR analysis was performed using a 10 μL reaction volume. Microcentrifuge tubes contained 0.6 μL of primer follistatin (*fst*) and 0.3 μL hepcidin (*hamp*), 5 μL of 2 × Eco, 1.7 μL of RNase/DNase free water, and 1.5 μL of the DNA sample before being run in a thermal cycler. Samples were run on a 2.0% agarose gel, using ethidium-bromide for staining, for the *fst* and *hamp* amplification products to be resolved (Waldbieser and Bosworth 2008; Hettiarachchi et al. 2020, 2023a, 2024; Table 1).

**Table 1:** The primers [*fst* (follistatin) and *hamp* (hepcidin antimicrobial protein)] that were used in PCR analysis to differentiate channel catfish (*Ictalurus punctatus*), blue catfish (*I. furcatus*), and white catfish (*Ameiurus catus*). Primers were previously described by Waldbieser and Bosworth (2008)

Gene	Forward primer	Reverse primer	Amplicon (bp)	
			Channel catfish	Blue catfish
*fst*	ATAGATGTAGAGGAGCATTTGAG	GTAACACTGCTGTACGGTTGAG	348	399
*hamp*	ATACACCGAGGTGGAAAAGG	AAACAGAAATGGAGGCTGGAC	222	262

### Statistical Analysis

SAS statistical analysis software (v.9.1; SAS Institute Inc., Cary, NC, USA) was used for data analyses. To ensure assumptions were met, residuals were tested for normality (Shapiro–Wilk test) and homogeneity of variance (plot of residuals vs. predicted values). Three-way ANOVA models were run to analyze survival, growth (BW and TL), and fluorescent imaging analyses (cell area and cluster area). ANOVA models contained cell density (80,000 and 100,000 cells/fry), sampling interval (45 and 90 DPH), and fry injection day (4-, 5-, and 6-DPH) main effects, and the associated interactions. If higher-order interactions were detected, the saturated models were decomposed into lower-order ANOVA models. If no significant interactions were detected, cell density, sampling interval, and fry injection day main effects were interpreted. When necessary, data was arcsin square root or log_10_ transformed to meet ANOVA assumptions. Alpha was set at 0.05 for main effects and interactions. Post-hoc testing was done using Tukey’s HSD test.

Fisher’s Exact Test was used to compare the percentage of xenogens detected using PCR compared to those detected using PKH26, and to evaluate potential differences in percentage of xenogens generated by blue catfish or channel catfish stem cells when injected into white catfish.

## Results

### Survival

**Fig. 5:**
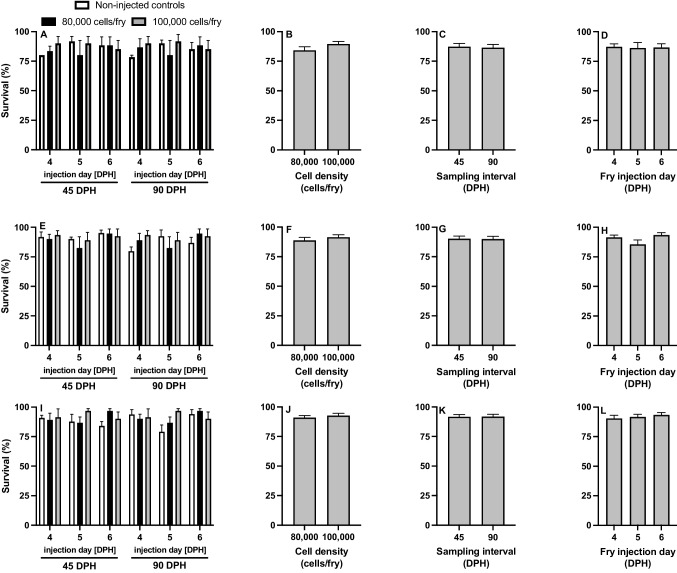
Percentage survival of **A**-**D** triploid xenogenic channel catfish (*Ictalurus punctatus*) injected with blue catfish (*I. furcatus*) unsorted gonadal cells, **E**–**H** triploid xenogenic white catfish (*Ameiurus catus*) injected with blue catfish unsorted gonadal cells, **I**-**L** triploid xenogenic white catfish injected with channel catfish unsorted gonadal cells with either **B**, **F**, **J** 80,000 or 100,000 cells/fry along with non-injected control treatment. (C, G, K) Fry were sampled at 45- and 90-days post-hatch (DPH). **D**, **H**, **L** Fry were injected at either 4, 5, or 6 DPH. No differences were found among means (*P* > 0.05, three-way ANOVA). *N* = 20 for the control for each day per donor experiment; N = 60 for each individual treatment (donor day × density); N = 360 for each cell density per donor; N = 360 for each sampling interval per donor; N = 240 for each injection day per donor

For all treatments, no significant interactions were detected for survival (Fig. 5; Table 2). Cell density (Fig. 5BFJ), sampling interval (Fig. 5CGK), and injection day (Fig. 5DHL) also had no impact on fry survival for all surrogate types. Survival of non-injected controls was not different from the fry injected with cells after 45 (*P* > 0.05) and 90 DPH (*P* > 0.05).

**Table 2:** Results of the analysis of variance (three-way ANOVA with interaction) for surrogate triploid channel catfish (*Ictalurus punctatus*) injected with blue catfish (*I. furcatus*) gonadal cells and triploid white catfish (*Ameiurus catus*) injected with channel catfish or blue catfish gonadal cells, respectively. Main effects include injection day (ID) (4, 5, 6, days post-hatch [DPH]), cell density (CD) (80,000 or 100,000 cells/fry), and sample day (SD) (45 and 90 DPH). (DFN = numerator degrees of freedom, DFD = denominator degrees of freedom, ƒ = ƒ value, *P* = p value, BW = body weight (g), TL = total length (cm), cell area < 150 μm^2^, cluster area > 150 μm^2^). Alpha was set at 0.05 for main effects and interactions

			*Survival*		*BW*		*TL*		*Cell area*		*Cluster area*	
Effect	DFN	DFD	*(f)*	*(P)*	*(f)*	*(P)*	*(f)*	*(P)*	*(f)*	*(P)*	*(f)*	*(P)*
Channel catfish surrogate, blue catfish gonadal cells												
Cell density (CD)	1	12	0.69	0.423	1.31	0.185	9.33	0.145	0.62	0.445	12.45	0.004
ID × CD	2	12	0.44	0.655	1.95	0.182	6.57	0.019	0.12	0.888	0.03	0.969
Sample day (SD)	1	12	0.65	0.437	6.20	0.001	7.44	0.001	10.47	0.007	3.25	0.096
ID × SD	2	12	2.12	0.162	0.55	0.006	0.063	0.833	0.06	0.938	0.08	0.923
ID × CD × SD	2	12	0.11	0.894	1.24	0.324	0.20	0.802	0.06	0.943	0.26	0.777
White catfish surrogate, blue catfish gonadal cells												
Cell density (CD)	1	12	0.17	0.684	0.43	0.53	0.1	0.76	3.39	0.091	11.87	0.005
Sample day (SD)	1	12	0.03	0.871	5.05	0.04	370	0.01	8.34	0.014	26.34	0.001
ID × CD × SD	2	12	0.03	0.973	0.58	0.58	0.08	0.93	0.08	0.923	0.44	0.654
White catfish surrogate, channel catfish gonadal cells												
Cell density (CD)	1	12	0.46	0.518	0.11	0.749	0.62	0.446	5	0.045	12.23	0.004
Sample day (SD)	1	12	0.03	0.871	14.21	0.003	757.15	0.001	19.07	0.001	19.17	0.001
ID × CD × SD	2	12	0.03	0.973	1.78	0.211	0.24	0.792	0.32	0.734	0.57	0.581

^*^Rows that had no significance are not listed except for the three-way interactions

### Growth Performance

**Fig. 6:**
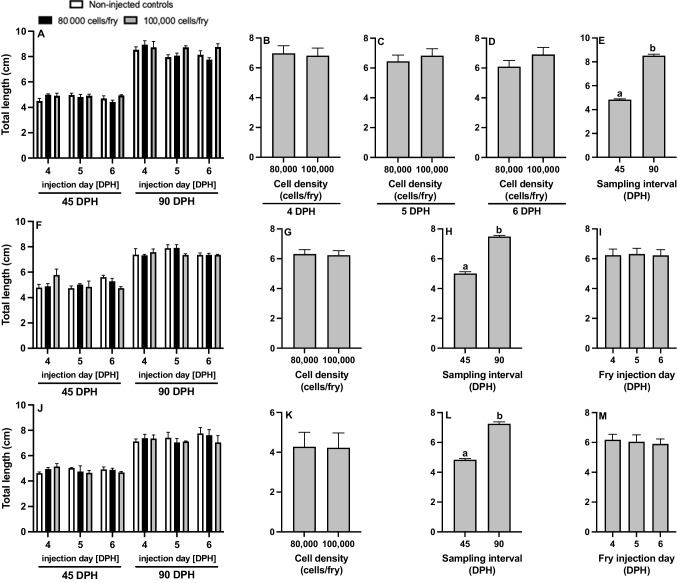
Total length (cm) of **A**-**E** triploid xenogenic channel catfish (*Ictalurus punctatus*) injected with blue catfish (*I. furcatus*) unsorted gonadal cells, **F**-**I** triploid xenogenic white catfish (*Ameiurus catus*) injected with blue catfish unsorted gonadal cells, **J**-**M** triploid xenogenic white catfish injected with channel catfish unsorted gonadal cells with either **B**-**D**, **G**, **K** 80,000 or 100,000 cells/fry along with non-injected control treatment. **E**, **H**, **L** Fry were sampled at 45- and 90-days post-hatch (DPH). **B**-**D**, **I**, **M** Fry were injected at either 4, 5, or 6 DPH. N = 9 for the control for each day per donor experiment; N = 27 for each individual treatment (donor day × density); N = 54 for cell density by day per donor; N = 162 for each cell density per donor; N = 162 for each sampling interval per donor; N = 108 for each injection day per donor.^ab^Means followed by the same letter are not different (*P* > 0.05, three-way ANOVA model)

For channel catfish BGC surrogates, fry TL had a significant injection day × cell density interaction (*P* = 0.019; Fig. 6A-E; Table 2). Therefore, the effect of injection day in relation to cell density was examined to understand the interaction (Fig. 6B-D). Cell density had no impact on fry TL at 4 (*P* = 0.910; Fig. 6B), 5 (*P* = 0.751; Fig. 6C), and 6 DPH (*P* = 0.504; Fig. 6D). At 90 DPH, fry were significantly longer than those sampled at 45 DPH (*P* = 0.001; Fig. 6E). TL of non-injected controls did not differ from that of the fry injected with cells after 45 (*P* > 0.05) and 90 (*P* > 0.05) days of growth. The mean BW of channel catfish and white catfish surrogates were 4.31 ± 0.50 g and 5.03 ± 0.50 g, respectively, at 45 DPH and 7.43 ± 0.60 g and 7.75 ± 0.47 g, respectively, at 90 DPH.

For white catfish BGC and white catfish CGC surrogates, no significant interactions were detected for TL (Fig. 6F-I; Fig. 6J-M; Table 2). Cell density (Fig. 6GK) and injection day (Fig. 6IM) had no impact on fry growth. Surrogates were significantly longer at the 90 DPH sampling interval compared to the 45 DPH interval (Fig. 6HL, Table 2). Non-injected control fry also had no performance differences from surrogates at 45 (*P* > 0.05) and 90 DPH (*P* > 0.05).

**Fig. 7:**
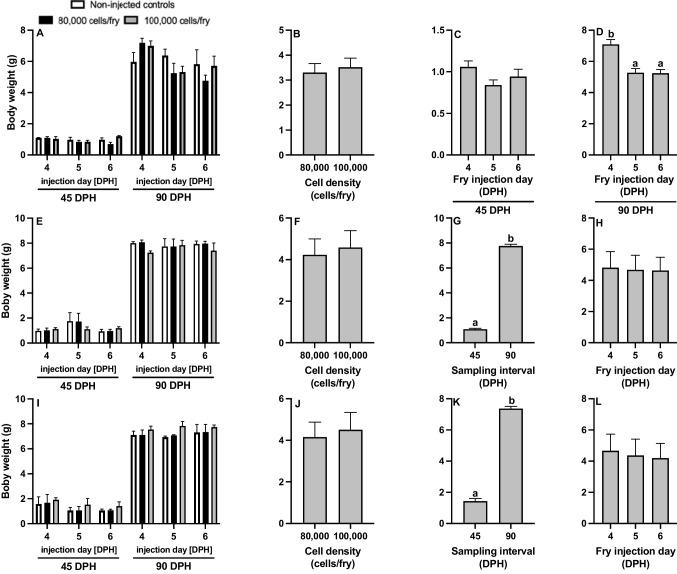
Body weight (BW) of **A**-**D** triploid xenogenic channel catfish (*Ictalurus punctatus*) injected with blue catfish (*I. furcatus*) unsorted gonadal cells, **E**–**H** triploid xenogenic white catfish (*Ameiurus catus*) injected with blue catfish unsorted gonadal cells, **I**-**L** triploid xenogenic white catfish injected with channel catfish unsorted gonadal cells with either **B**, **F**, **J** 80,000 or 100,000 cells/fry (N = 63) along with non-injected control treatment. **C**, **G**, **K** Fry were sampled at 45- and 90-days post-hatch (DPH). **D**, **H**, **L** Fry were injected at either 4, 5, or 6 DPH. N = 9 for the control for each day per donor experiment; N = 27 for each individual treatment (donor day × density); N = 54 for cell density by day per donor; N = 162 for each cell density per donor; N = 162 for each sampling interval per donor; N = 108 for each injection day per donor. ^ab^Means followed by the same letter are not different (*P* > 0.05, three-way ANOVA model)

For BW of channel catfish BGC surrogates, the injection day × sampling interval interaction was significant (*P* = 0.006; Fig. 7A-D; Table 2); therefore, the model was revised to examine the effect of injection day at each sampling interval (Fig. 7CD). At 45 DPH, BW did not differ between the injection days (*P* = 0.482; Fig. 7C), while at 90 DPH, fry injected on 4 DPH were significantly heavier than those injected on 5 or 6 DPH (*P* = 0.001; Fig. 7D). Cell density had no impact on fry BW (*P* = 0.185, Fig. 7B). BW of non-injected controls did not differ from that of the fry injected with cells after 45 (*P* > 0.05) and 90 (*P* > 0.05) days of growth.

No significant interactions were detected for BW of white catfish BGC and white catfish CGC surrogates (Fig. 7E-H; Fig. 7I-L; Table 2). Cell density (Fig. 7FJ) and injection day (Fig. 7HL) also had no impact on fry BW. Fry were significantly heavier (Fig. 7GK) by the 90 DPH sampling interval. Non-injected control fry also had no performance differences from surrogates at 45 and 90 DPH (*P* > 0.05).

#### Quantifying PKH26 Florescent Labeling

**Fig. 8:**
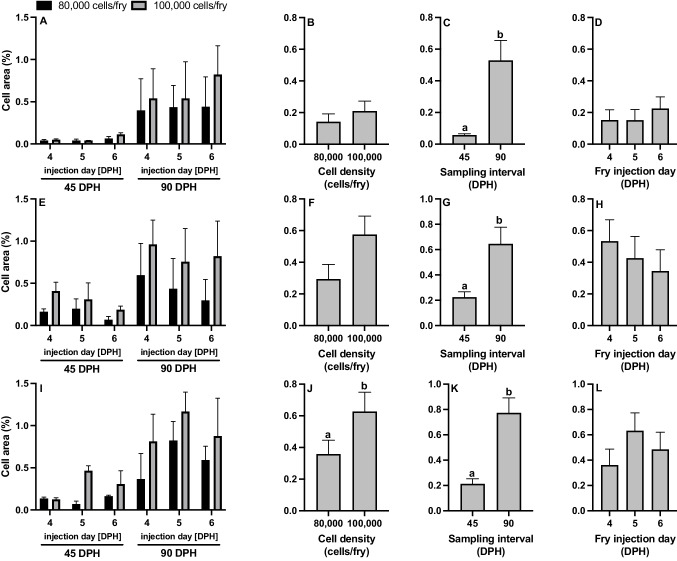
Cell area (%, < 150 μm^2^) of **A**-**D** triploid xenogenic channel catfish (*Ictalurus punctatus*) injected with blue catfish (*I. furcatus*) unsorted gonadal cells, **E**–**H** triploid xenogenic white catfish (*Ameiurus catus*) injected with blue catfish unsorted gonadal cells, **I**-**L** triploid xenogenic white catfish injected with channel catfish unsorted gonadal cells with either **B**, **F**, **J** 80,000 or 100,000 cells/fry (N = 63). (C, G, K) Fry were sampled at 45- and 90-days post-hatch (DPH). **D**, **H**, **L** Fry were injected at either 4, 5, or 6 DPH. Non-injected controls violated ANOVA assumptions and were excluded from the analysis. For each host-donor combination: N = 9 for the control for each day per donor experiment; N = 9 for each individual treatment (donor day × density); N = 54 for cell density; N = 54 for each sampling interval; N = 36 for each injection day. ^ab^Means followed by the same letter are not different (*P* < 0.05, three-way ANOVA model) and negative individuals were included in the calculation of the mean

Unsorted gonadal cell colonization and proliferation was quantified using PKH26 dye (Fig. 4). There were no significant interactions for all surrogate types when analyzing percent cell area (Fig. 8; Table 2). Cell density did not have an impact on colonization or proliferation rates in fry for percent cell area of channel catfish BGC surrogates, white catfish BGC surrogates, and white catfish CGC surrogates, but percent cell area was found to be significantly larger in white catfish CGC surrogates injected with 100,000 cells/fry compared to those injected with 80,000 cells/fry (Fig. 8J; Table 2). Percent cell area increased significantly from 45 to 90 DPH for all surrogate types (Fig. 8CGK) whereas injection day had no impact on percent cell area (Fig. 8DHL).

**Fig. 9:**
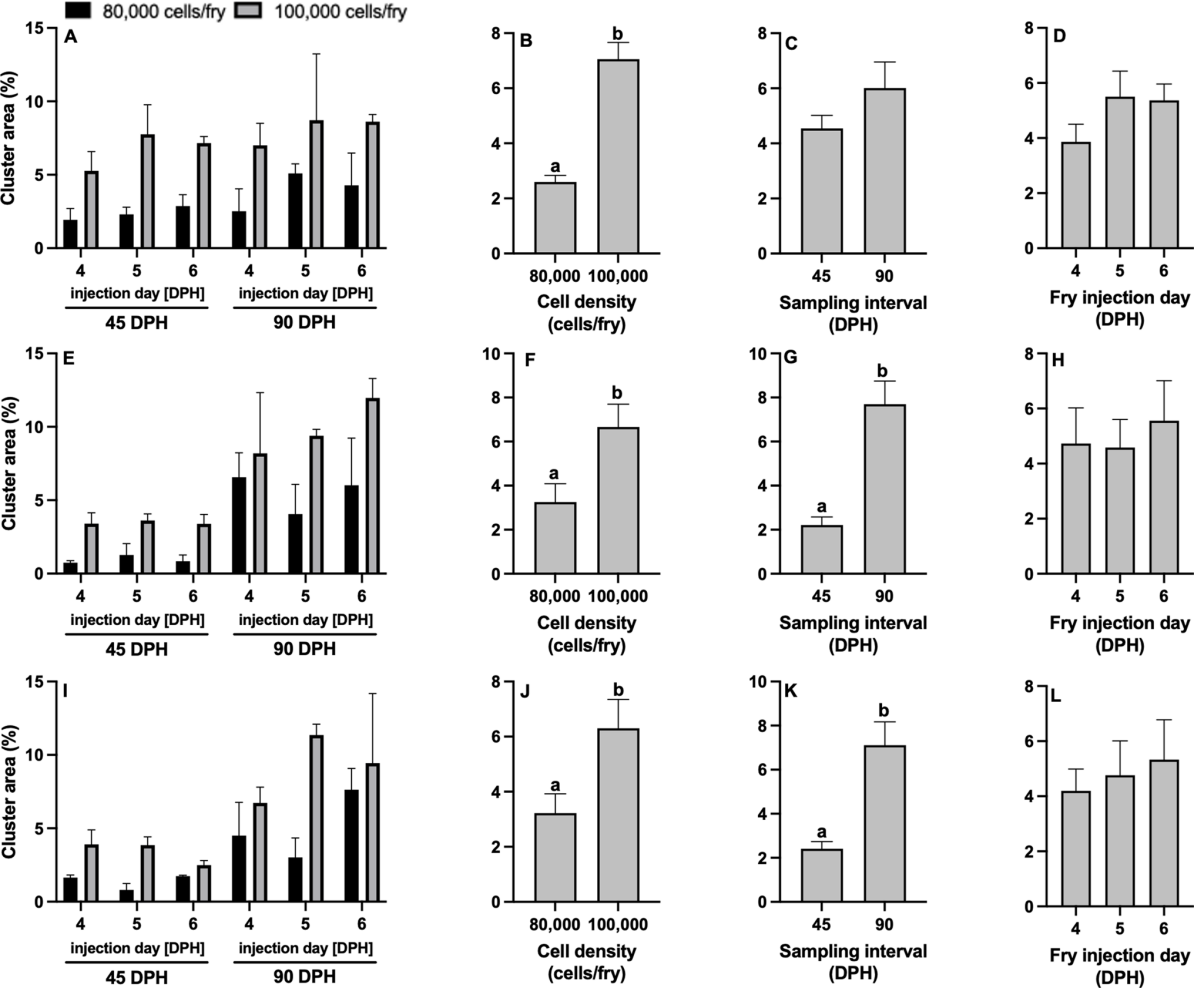
Cluster area (%, > 150 μm^2^) of **A**-**D** triploid xenogenic channel catfish (*Ictalurus punctatus*) injected with blue catfish (*I. furcatus*) unsorted gonadal cells, **E**–**H** triploid xenogenic white catfish (*Ameiurus catus*) injected with blue catfish unsorted gonadal cells, **I**-**L** triploid xenogenic white catfish injected with channel catfish unsorted gonadal cells with either **B**, **F**, **J** 80,000 or 100,000 cells/fry. **C**, **G**, **K** Fry were sampled at 45- and 90-days post-hatch (DPH). **D**, **H**, **L** Fry were injected at either 4, 5, or 6 DPH. Non-injected controls violated ANOVA assumptions and were excluded from the analysis. For each host-donor combination: N = 9 for the control for each day per donor experiment; N = 9 for each individual treatment (donor day × density); N = 54 for cell density; N = 54 for each sampling interval; N = 36 for each injection day. ^ab^Means followed by the same letter are not different (*P* < 0.05, three-way ANOVA model) and negative individuals were included in the calculation of the mean

Percent cluster area also had no significant interactions for any surrogate type (Fig. 9; Table 2). Cell density had a significant impact on colonization and proliferation rates in all surrogate fry types for percent cluster area (Fig. 9BFJ), such that 100,000 cells/fry led to increased fluorescing regions in surrogates. Sampling interval (Fig. 9C) had no impact on channel catfish BGC surrogates, but for white catfish BGC and white catfish CGC surrogates, percent cluster area increased in size from the 45 to 90 DPH sampling interval (Fig. 9GK). Fry injection day had no impact on percent cluster area for any surrogate type (Fig. 9DHL). Non-injected controls were found to have no cell area or cluster area at 45 and 90 DPH.

### Donor Species Comparison

Survival, growth performance, and fluorescent labeling data for surrogate white catfish surrogates injected with BGCs were compared to surrogates injected with CGCs to assess the viability of each donor species. At the first and second sampling intervals, no differences were observed among surrogates injected with BGCs or CGCs and between 80,000 or 100,000 cells/fry for survival, TL, BW, cell area, and cluster area.

### Xenogen Detection

**Fig. 10:**
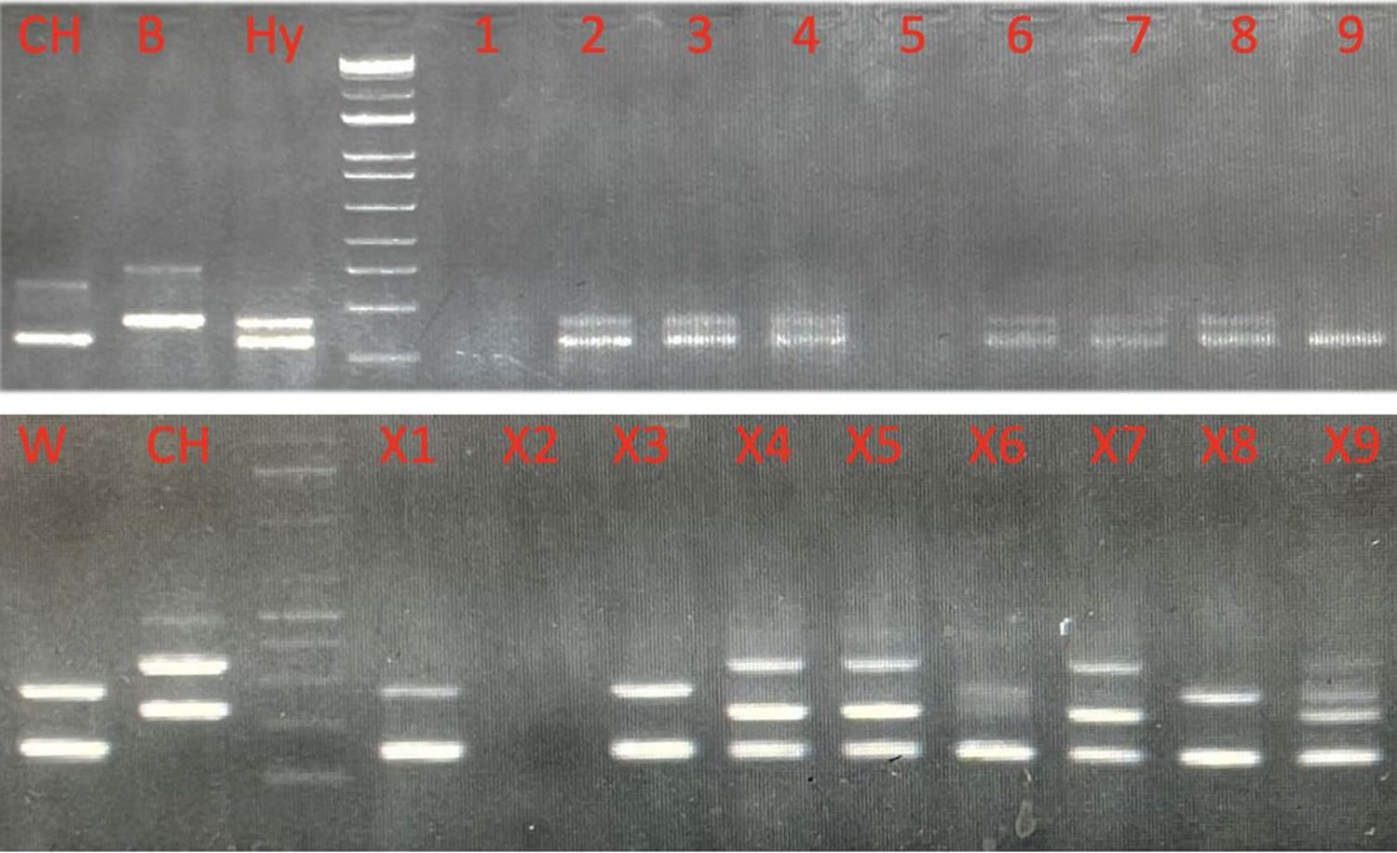
(Top gel; 1–9) Sample results from PCR for detecting blue catfish (*Ictalurus furcatus*) donor cells in the gonads of triploid channel catfish (*I. punctatus*). Individuals 2, 3, 4, 6, 7, and 8 are xenogens as they show a hybrid catfish banding pattern (blue catfish marker from stem cells and channel catfish marker from somatic cells). (Bottom gel; X1-X9) Sample results from PCR for detecting channel catfish in triploid white catfish (*Ameiurus catus*) gonads. Individuals 4, 5, 7, and 9 are xenogens as they show channel catfish stem cell markers and white catfish host somatic markers. Blue catfish, channel catfish, and white catfish cells were differentiated with PCR using follistatin (*fst*) and hepcidin antimicrobial protein (*hamp*) genes as markers. CH = channel catfish control, B = blue catfish control, W = white catfish control, Hy = female channel catfish × male blue catfish hybrid control

PCR analysis detected blue catfish donor-derived cells in the gonads of triploid channel catfish fry when injected with 80,000 and 100,000 cells/fry (Fig. 10). When channel catfish BGC surrogates were observed at 45 DPH, the percentage of xenogens detected was 83.7% and 79.3% for surrogates injected with 80,000 and 100,000 cells/fry, respectively. At 90 DPH, the percentage of xenogens detected was 77.78% and 66.67% for channel catfish BGC surrogates injected with 80,000 and 100,000 cells/fry, respectively. No significant differences were found when comparing percent xenogens detected by DNA analysis for channel catfish BGC surrogates injected with 80,000 cells/fry vs. 100,000 cells/fry (*P* = 0.260).

Percent xenogens also were not different (*P* < 0.999) for channel catfish BGC surrogates injected with 80,000 cells/fry (100.0%) vs. 100,000 cells/fry (94.4%) as detected by PKH26 analysis. Significantly more (*P* = 0.010) channel catfish BGC xenogens were detected using PKH26 (97.2%) than with PCR (77.8%).

PCR analysis indicated transplanted BGCs and CGCs were present in the gonads of surrogate white catfish fry during the 45 and 90 DPH sampling intervals (Fig. 10). At 45 DPH, the percentage of white catfish BGC xenogens was 77.8% and 88.9% when recipients were injected with 80,000 and 100,000 cells/fry, respectively (*P* = 0.543). The percentage of white catfish CGC xenogens at 45 DPH was 77.78% for both 80,000 and 100,000 cells/fry (*P* = 0.999). The percentage xenogens using BGCs (grand mean of 86.5%, N = 216) and CGCs (grand mean of 86.0%, N = 216) were not different in white catfish surrogates for all treatment parameters.

At 90 DPH, the percentage of white catfish BGC xenogens was 83.3% and 91.7% when recipients were injected with 80,000 and 100,000 cells/fry, respectively (*P* = 0.792). The percentage of white catfish CGC xenogens at 90 DPH was 75.0% and 83.3% for 80,000 and 100,000 cells/fry, respectfully (*P* = 0.455) (Fig. 8).

Percent xenogens were not different (*P* = 0.104) for white catfish BGCs surrogates injected with 80,000 cells/fry (77.8%) vs. 100,000 cells/fry (100%) as detected by PKH26 analysis. Similarly, percent xenogens were not different (*P* < 0.999) for white catfish CGCs surrogates injected with 80,000 cells/fry (88.8%) vs. 100,000 cells/fry (88.8%). No significant (*P* = 0.503) differences were found among xenogens detected using PKH26 (86.1%) vs. PCR (81.9%).

## Discussion

The present study is in accordance with recent findings by Hettiarachchi et al. (2024), showing the white catfish as a feasible surrogate, incorporating both BGCs and CGCs donor cells, along with the channel catfish surrogate. The present study also demonstrates that survival and growth of xenogens were not impacted by injecting higher quantities of unsorted gonadal cells and 100,000 cells/fry leads to increased percent cluster area colonization and proliferation in xenogenic catfish surrogates. As well, both percentage of cell and cluster area increased from 45 to 90 DPH, demonstrating increased rates of proliferation. Channel catfish and white catfish appear equally effective as hosts to produce xenogenic fry for future utilization as broodstock to produce channel catfish female × blue catfish male hybrid catfish embryos. These results add to the current body of knowledge on xenogenesis for catfish (Perera et al. 2017; Hettiarachchi et al. 2020, 2022, 2023a, b) along with past studies using other surrogate species (Morita et al. 2015; Lujić et al. 2018; Franěk et al. 2021, 2022).

Utilization of xenogenic catfish can improve hybrid catfish hatchery efficiency. However, increasing the number of injected cells inside the surrogate could lead to increased mortality, as transplantation of cells into sterile fry can often result in injury and stress (Hettiarachchi et al. 2023a). As such, mortalities are likely to occur as the number of cells injected into the surrogate increases, especially due to the sensitivity of fry during these “critical” early life stages. However, we found no differences in survival between fry injected with 80,000 or 100,000 cells, suggesting that 100,000 cells/fry can be utilized for increasing xenogen output.

In this study, we used percent cell area and cluster area to quantify colonization and proliferation rates of injected cells in surrogate gonads. Past studies using various forms of surrogacy applications have used 5,000 spermatogonia cells/fry in germ cell-depleted zebrafish (*Danio rerio*) surrogates (Franěk et al. 2022), ~ 15,000 germline stem cells/fry in rainbow trout (*Oncorhynchus mykiss*) surrogates (Marinović et al. 2022), and 30,000 to 50,000 germ stem cells/fry in goldfish (*Carassius auratus*) surrogates (Franěk et al. 2021). Thus, the number of cells/fry varies based on species. In our experiments only ~ 50 to 60% of injected unsorted gonadal cells are SSCs and OSCs (Perera et al. 2017; Shang et al. 2018). Despite this, our injection density is still at the higher end of the spectrum, as compared to most aquatic species. Injecting germ stem cells in surrogates is the most common method for creating xenogens, but cell extraction protocols vary by species (Lujić, et al. 2018; Hettiarachchi et al. 2023a; Franěk et al. 2021, 2022; Marinović et al. 2022). A universal protocol is not feasible as species morphology, physiology, and development differs.

In preliminary studies, we attempted to inject 120,000 cells/fry. However, needle clogs (outer diameter: 0.209 mm; inner diameter: 0.108 mm) occurred frequently and successful injection was not possible (unpublished data). This prevented us from testing cell injection quantities > 100,000 cells/fry. A larger needle gauge (outer diameter: 0.261 mm; inner diameter: 0.133 mm) yielded fewer clogs but resulted in large puncture wounds in the fry, often piercing through the body cavity. Thus, at present, 100,000 cells/fry appears to be the upper injection threshold, until more suitable injection techniques become available.

In the current study, cell area percentage increased as surrogate fry aged. This aligns with past studies by Hettiarachchi et al. (2023a), demonstrating successful colonization and continued proliferation of cells in surrogates. In other species, colonization and migratory potential are strongly influenced by injection age, often decreasing as the age at injection increases (Franěk et al. 2022). In channel catfish surrogates, injecting cells after 7 DPH decreased colonization success (Hettiarachchi et al. 2023a). Hettiarachchi et al. (2023a) found the optimal injection days for channel catfish fry to be 4 to 6 DPH. Percent cell area in the surrogates decreased slightly (~ 5 to 4%) from the first to the second sampling interval, but percent cluster area increased in size at the second sampling interval (~ 4 to 9%), likely due to cell areas combining to create cluster areas (Hettiarachchi et al. 2023a). A similar study by Hettiarachchi et al. (2022) using xenogenic channel catfish, also found both percent cell area and cluster area to increase from the first to the second sampling interval, as was found in the current study, when using both fresh and cryopreserved SSCs and OSCs (Hettiarachchi et al. 2022). Across other species, there are inconsistencies whether injected cell colonies tend to increase or decrease as surrogate fry age, as the biggest factor tends to be related to surrogate acceptance of the foreign cells (Franěk et al. 2022). Of course, sampling time and the eventual fading of the fluorescent dye are variables that can have a strong impact on the variation among these studies.

Future research for producing xenogenic catfish should focus on utilizing pure populations of stem cells to reduce the number of cells injected. This would require some type of purification procedure. As stated previously, to produce xenogenic catfish ~ 50 to 60% of injected cells are stem cells (Perera et al. 2017; Shang et al. 2018). Thus, the number of somatic cells injected could be reduced by improving in vitro pure stem cell culture. This would likely improve colonization and proliferation rates in the gonad region and reduce the need for higher quantities of unwanted cells being injected into surrogates. Current cell purification techniques for catfish stem cells are inefficient and lead to excessive cell death (Hettiarachchi et al. 2022). Thus, improving cell culture to generate sustained cell growth, development, and regeneration could also reduce the number of immature donor fish sacrificed. Bhattarai et al. (2023) have successfully cultured (in vitro) black crappie (*Pomoxis nigromaculatus)* and white crappie (*P. annularis*) ovarian tissue primary cells. Similar cell culture efforts with the blue catfish would be beneficial for the creation of the xenogenic catfish.

Additionally, we showed that neither donor species (blue catfish or channel catfish) for the creation of the xenogenic white catfish were superior to the other. This is an important result, especially if the goal is to mate xenogenic white catfish females producing channel catfish eggs with xenogenic white catfish males producing blue catfish sperm to produce hybrid progeny. Due to favorable characteristics of white catfish, such as early sexual maturity and good spawning rates, incorporation of this species into xenogenic technology could be a pivotal change for the hybrid catfish industry. The demonstration that the white catfish surrogate readily accepts BGCs and CGCs donor cells adds further validation of using white catfish to produce channel-blue hybrid catfish embryos (Hettiarachchi et al. 2024).

The surrogate’s acceptance of foreign donor cells could be an obstacle for using some species for xenogenic technology development. Some surrogate species may reject foreign cells (Lee and Yoshizaki 2016). If surrogate acceptance is achieved and colonization is confirmed, genetic tools such as selective breeding can be used to further advance a surrogate’s performance (Yoshizaki and Yazawa 2019). Selection for the best phenotypes and genotypes could take place for both immature donor and juvenile surrogate fish, though surrogate selection may be more difficult depending on the optimal DPH for injection (Yoshizaki and Yazawa 2019). Using specific DNA markers can enable simpler and quicker identification of ideal phenotypes in larvae fry and donor juveniles (Abdelrahman et al. 2017). Incorporating both host specification and selective breeding, along with maternal and paternal host genetics, nutrition, and environmental impacts, into xenogenesis can not only enhance efficiency but also sustainability within the technology.

Utilizing surrogate species with shortened generation times for increased efficiency has been explored within other aquaculture systems (Ryu et al. 2022). For example, chinook salmon (*Oncorhynchus tshawytscha*) females take 3 years and males take 5 years to reach sexual maturity. Rainbow trout females can reach sexual maturity in 2–3 years and males in 1–2 years. Using xenogenesis to produce chinook salmon germ cells in surrogate rainbow trout larvae can shorten generation time and enhance production (Ryu et al. 2022).

The white catfish presents a unique opportunity for potential adoption of xenogenesis in the catfish industry with its shortened time to maturation, smaller body size and spawning early in the breeding season. The culture requirements of white catfish have been widely studied and are similar to that of channel catfish (Prather and Swingle 1960; Prather 1964; Greene 1971; Perry and Avault 1968, 1969; Green 1973; Loyacano 1974; Chappell 1979; Fobes 2013), and although channel catfish eventually became the food fish of choice for culture, white catfish have reproductive traits that could make them a good surrogate to produce hybrid catfish (Hettiarachchi et al. 2024). The entire catfish industry has not adopted the hybrid catfish. Xenogenesis can also benefit other sectors of the catfish industry. Xenogenic white catfish could be used to accelerate selective breeding programs for both channel catfish and blue catfish by reducing the generation interval as well as speeding reciprocal recurrent selection to produce enhanced hybrid progeny. Natural spawning and particularly induced hormone spawning of blue catfish is erratic compared to channel catfish. One application of xenogenesis is spawning of both difficult to spawn and late maturing fish such as blue catfish.

There are some potential drawbacks to utilizing the white catfish for xenogenesis (Hettiarachchi et al. 2024). In pond culture, white catfish broodstock have lower survival, seinability, and fighting issues (especially among males), which can lead to injuries and secondary infections (Fobes 2013). This is also problematic in channel catfish, particularly male fighting and survival, although our preliminary data indicates to a slightly lesser extent compared to white catfish. One way to avoid these challenges with the white catfish is to use recirculating aquaculture systems (RAS) for culture of the broodstock instead of pond culture. RAS allows for a more controlled setting such as stocking density control, incorporation of shelters to alleviate fighting, and eliminates harvesting issues and is relatively sustainable compared to other concentrated animal feeding operations (Hettiarachchi et al. 2024). Our preliminary data also indicates that preparing both channel catfish and white catfish in flow through tanks may be a solution to broodstock aggression.

In conclusion, increasing gonadal cell quantities did not impact fry survival and growth but did enhance colonization and proliferation rates. The white catfish is a suitable candidate for xenogenesis application with both BGCs and CGCs being accepted by surrogates. Over time, cell proliferation was higher when 100,000 cells were injected compared to 80,000 cells. These findings are important for advancing the hybrid catfish industry along with development of xenogenesis that is technically and economically feasible for commercial use. Continued efforts to further establish the technology should be made, especially as the aquaculture industry continues to grow in the U.S. Enhancing purity of donor cells will be a crucial next step in xenogenesis technology for ictalurid catfish to further improve efficiency, sustainability, and feasibility of this system.

## Supplementary Information

Below is the link to the electronic supplementary material.

Supplementary file1 (DOCX 3250 KB)

## Data Availability

Data utilized in this study will be made available upon request.
