# Supplementary material for: Impacts of Host, Cell Density, and Timing of Injection on Efficiency for Xenogen Production in Ictalurid Catfish
Source: Mar Biotechnol (NY). 2025 May 27;27(3):90. doi: 10.1007/s10126-025-10466-5 (PMC12117003; doi:10.1007/s10126-025-10466-5)
Supplement: Supplementary file 1 — Supplementary file1 (DOCX 3250 KB) [file 10126_2025_10466_MOESM1_ESM.docx]

**Figure 10 – Full, uncropped, gel full images**

First image below is the top half (1-9) of Figure 10.

Second image below is the bottom half (X1-X9) of Figure 10.

**
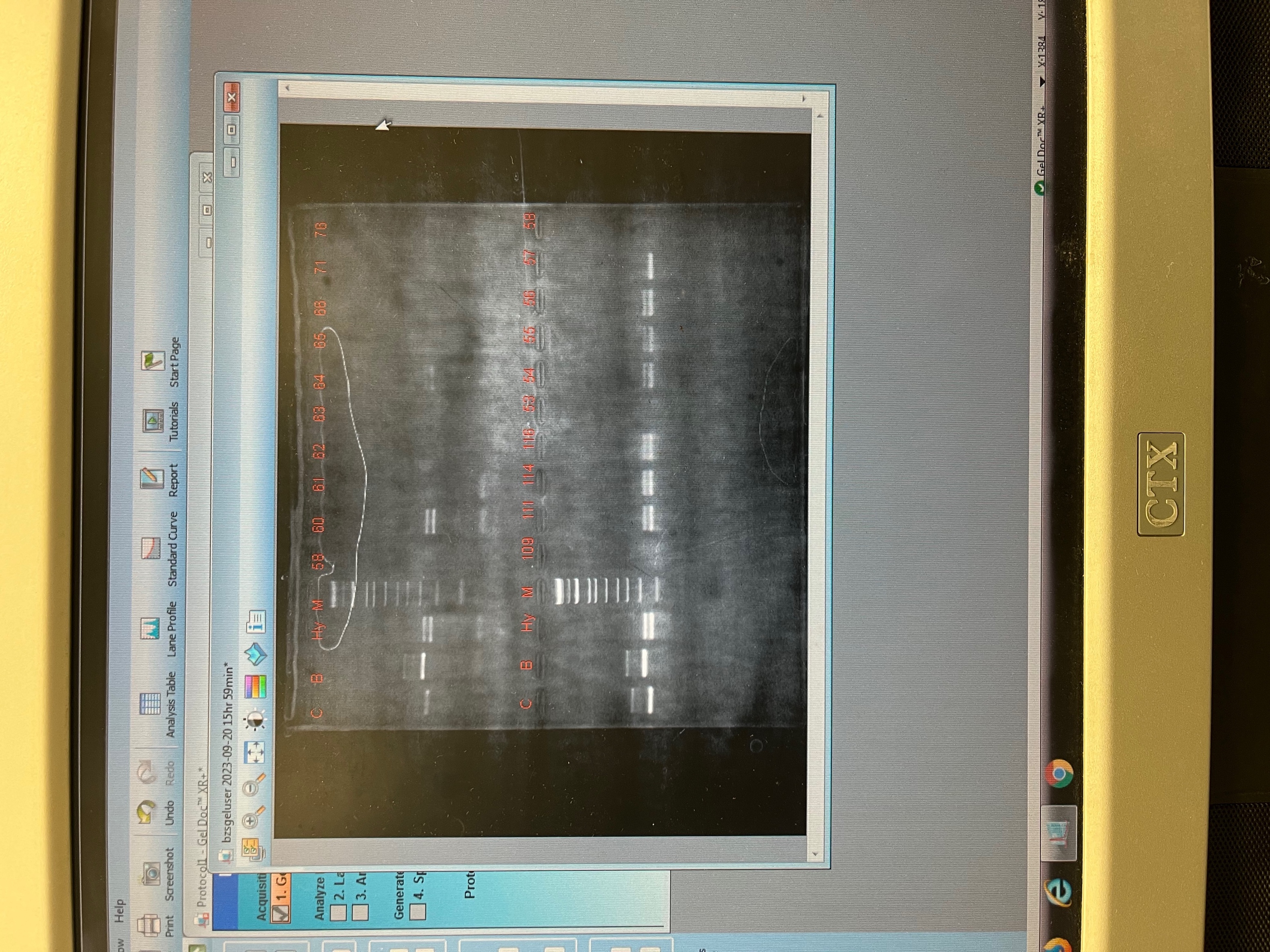
**

- Figure 10. (1-9)
- Cropped to use the bottom half of this gel image to improve viewing quality
- Cropped out the original numbers I added to improve clarity to the manuscript by adding a concise numbering system


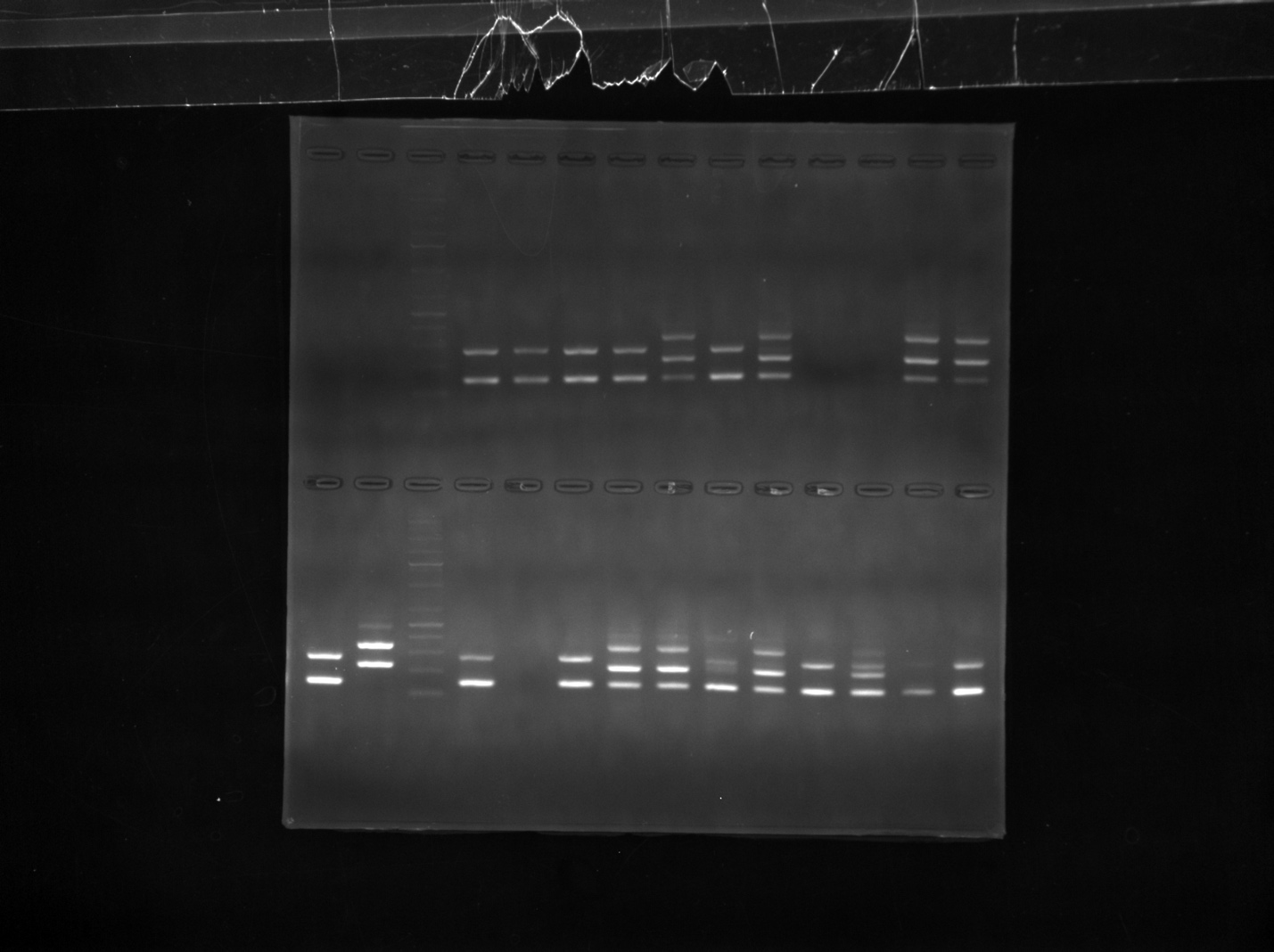


- Figure 10. (X1-X9)
- Cropped to use the bottom half of this gel image to improve viewing quality
- I added numbering to add clarity to the manuscript by incorporating concise numbering system
